# Comparing early years and childhood experiences and outcomes in Scotland, England and three city-regions: a plausible explanation for Scottish ‘excess’ mortality?

**DOI:** 10.1186/1471-2431-14-259

**Published:** 2014-10-10

**Authors:** Martin Taulbut, David Walsh, John O’Dowd

**Affiliations:** NHS Health Scotland, Meridian Court, 5 Cadogan Street, Glasgow, Scotland; Glasgow Centre for Population Health, House 6, 94 Elmbank Street, Glasgow, Scotland; University of Glasgow, 1 Lilybank Gardens, Glasgow, Scotland; NHS Ayrshire and Arran Health Board, Afton House, Dalmellington Road, Ayr, Scotland

**Keywords:** Child health, Spatial analysis, Social and life-course epidemiology

## Abstract

**Background:**

Negative early years and childhood experiences (EYCE), including socio-economic circumstances, parental health and parenting style, are associated with poor health outcomes both in childhood and adulthood. It has also been proposed that EYCE were historically worse in Scottish areas, especially Glasgow and the Clyde Valley, compared to elsewhere in the UK and that this variation can provide a partial explanation for the excess of ill health and mortality observed among those Scottish populations.

**Methods:**

Multiple logistic regression analysis was applied to two large, representative, British birth cohorts (the NCDS58 and the BCS70), to test the independent association of area of residence at ages 7 and 5 with risk of behavioural problems, respiratory problems and reading/vocabulary problems at the same age. Cohort members resident in Scotland were compared with those who were resident in England, while those resident in Glasgow and the Clyde Valley were compared with those resident in Merseyside and Greater Manchester.

**Results:**

After adjustment for a range of relevant variables, the risk of adverse childhood outcomes was found to be either no different, or lower, in the Scottish areas. At a national level, the study reinforces the combined association of socio-economic circumstances, parental health (especially maternal mental health) and parenting with child health outcomes.

**Conclusion:**

Based on these samples, the study does not support the hypothesis that EYCE were worse in Scotland and Glasgow and the Clyde Valley. It seems, therefore (based on these data), less likely that the roots of the excess mortality observed in the Scottish areas can be explained by these factors.

**Electronic supplementary material:**

The online version of this article (doi:10.1186/1471-2431-14-259) contains supplementary material, which is available to authorized users.

## Background

Early years’ and childhood experiences (EYCE), including socio-economic circumstances, parental health and parenting style, play an important role in determining childhood outcomes, especially social, emotional and mental health, physical health and learning and development. Risk of childhood behavioural problems is increased by factors such as poverty, low educational attainment and housing difficulties, smoking in pregnancy and maternal malaise, as well as low perceived parenting skills [1–4]. Physical health in childhood, including limiting long-term illness and asthma, is associated with poor maternal physical health and low household income [5, 6]. Children’s cognitive skills (e.g. vocabulary, visual-motor coordination) are also highly correlated with household income [7] as well as maternal educational attainment, poor maternal health, early motherhood and aspects of parenting [6].

EYCE and their associated outcomes can have consequences for health later in life [8]. Beginning with the *experiences* themselves, analysis of British birth cohort data has found that childhood material disadvantage (such as paternal social class and living in social housing) increases the risk of poor self-reported health, smoking and premature mortality in adulthood [9, 10]. Other research has found that people reporting poor relationships with their parents in childhood and adolescence are more likely to report three or more health problems in adulthood and, in the case of women, have poorer psychological well-being [11, 12]. Exposure to adverse child experiences (such as abuse or neglect), problem behaviour in childhood and parental disinterest in their child’s education when the child was aged 11, also increases the relative risk of premature death [13, 14].

Turning to the *outcomes* arising from EYCE, childhood social, emotional and mental health (especially conduct disorders) and psychological health disorders have also been found to be associated directly with increased risk of poor adult health, including smoking and worse mental and physical health, in adulthood [15, 16]. They are also associated with adult determinants of poor health, such as lower educational attainment, reduced economic participation and earnings, relationship difficulties, financial problems and being in trouble with the law [16, 17].

Poor childhood physical health appears to increase the risk of poor self-reported general health, respiratory problems and depression in early adulthood [18] and chronic health conditions in older working-age adults (aged 55–65) [19]. In addition to the direct association, childhood morbidity is associated with lower educational attainment and earnings in adulthood [20], which may make a further contribution to adult ill-health.

Finally, childhood cognitive development is associated both indirectly and indirectly with adult health. Lower levels of cognitive development at age seven have been shown to be associated with increased risk of chronic illnesses in adulthood [21]. Higher levels of childhood cognitive development can also lower the risk of poor mental health in adulthood for women (although it may also increase the risk of alcohol abuse for both genders) [22]. Improved childhood learning and development outcomes can also protect adult health indirectly through their association with higher levels of educational attainment, and lower risk of both economic inactivity (for men only) and receipt of welfare benefits in middle-age [23].

Negative early years’ and childhood experiences have also been proposed as a possible influence on the ‘excess’ levels of poor health seen in Scotland, especially in Glasgow and the West Central Scotland conurbation [24]. This ‘excess’ relates to the higher levels of mortality seen in Scottish areas, even after controlling for age, sex and deprivation. It has been shown to exist for Scotland compared with England [25] and more recently for Glasgow compared with Liverpool and Manchester, where despite these cities sharing identical deprivation profiles, premature mortality is 30% higher in the Scottish city [26]. Geography of residence (in Scotland compared to England, and Glasgow and surrounds compared to similar English conurbations) therefore seems an important independent influence on rates of adult morbidity and mortality, though the factors driving this difference remain unclear.

Following publication of these studies, a number of hypotheses were proposed, ranging from ‘upstream’ (e.g. social and economic inequality, deindustrialisation) to ‘downstream’ explanations (e.g. differences in health behaviours or individual values) [27]. Early years and childhood factors, especially family breakdown, acrimony between partners or dysfunctional parenting, were included among these hypotheses as a ‘midstream’ explanation. Although these are hard to measure directly, we can measure childhood health and social outcomes associated with negative early years and childhood factors, and include geography of residence alongside wider determinants of health. If this theory is plausible, it might be expected that living in Scottish areas in childhood would also be associated with increased risk of poor childhood outcomes, after adjustment for a range of other relevant variables.

In 2013, the Glasgow Centre for Population Health published a report comparing early years’ and childhood experiences in Scotland, England and three ‘city-regions’ (Glasgow & the Clyde Valley (GCV), Greater Manchester (GM) and Merseyside) [28]. Its purpose was to investigate whether there were differences in these health determinants that might help explain the poor health of Scotland and GCV relative to these areas. Few clear differences in contemporary childhood and early years’ experiences emerged from the analyses. The exceptions were smoking during pregnancy and breastfeeding at a national level, although at a regional level the size of these differences diminished or even disappeared. There were also more ambiguous findings on dysfunctional households, parental warmth and shouting at children. However, the study was descriptive only and did not test for the influence of multiple factors simultaneously on childhood health outcomes.

This paper fills a gap in the knowledge base by formally testing whether historic early years’ and childhood experiences were worse in Scotland and GCV compared to other places, all other things being equal. It does this by using more sophisticated statistical methods –multiple logistic regression analysis – to examine the association between geography of residence and child health outcomes, while controlling for a range of important social, economic and family characteristics.

The hypothesis is that the early years’ experiences (in particular child poverty, parenting, or some combination of these) for children growing up in Scotland and West Central Scotland was worse compared to children growing up in England, Merseyside and Greater Manchester. This led to poor childhood outcomes, which in turn fed through to poorer adulthood health and higher rates of morbidity and premature mortality. In this phase of research the focus is on exploring whether or not residence of Scottish areas in childhood resulted in poorer childhood health outcomes, even after taking other factors into account.

## Methods

Two large cohort studies, the National Child Development Study (NCDS58) and the British Cohort Study 1970 (BCS70), were used to test the hypotheses. It was decided not to use the Millennium Cohort Study (MCS), despite its more comprehensive measures of parenting and health, because its Merseyside sample largely excludes Liverpool City residents and was therefore considered unrepresentative. Essential to this research, both the NCDS58 and BCS70 record cohort members’ area of residence (at a national and sub-regional level) at each sweep [29]. Informed consent was obtained from the parents of cohort members for childhood measurements. NCDS58 and BCS70 data are open access datasets available to non-profit research organisations.

### Participants

Only cohort members resident in Scotland or England were selected for the national analyses, providing 14,585 cases in the NCDS58 and 12,323 in the BCS70. For regional comparisons, only cohort members living in Glasgow & the Clyde Valley, Greater Manchester or Merseyside at ages 7 (NCDS58) and 5 (BCS70) were chosen, with a total of 1,502 NCDS58 cases and 1,247 BCS70 cases. Tables 1 and 2 provide descriptive statistics on the original datasets.

**Table 1:** **Descriptive statistics for relevant explanatory and outcome variables, NCDS 1958**

	N	%		N	%
*Explanatory variables*					
**Area of residence (aged 7)**					
England	12945	88.8	GCV	701	46.7
Scotland	1640	11.2	Merseyside	399	26.6
			Gr. Manchester	402	26.8
**Social class of father**					
Class 1 & 2	2311	15.8	Class 1 & 2	174	11.6
Class 3	8140	55.8	Class 3	820	54.6
Class 4 & 5	2890	19.8	Class 4 & 5	399	26.6
Missing	1244	8.5	Missing	109	7.3
**Low birth weight**					
Not LBW	9761	66.9	Not LBW	966	64.3
LBW	1049	7.2	LBW	110	7.3
Missing	3775	25.9	Missing	426	28.4
**Mother in school after MLA**					
Stayed	3455	23.7	Stayed	230	15.3
Not stayed	10553	72.4	Not stayed	1238	82.4
Missing	577	4.0	Missing	34	2.3
**Housing tenure**					
Owner occupied	5775	39.6	Owner occupied	453	30.2
Rented	7998	54.8	Rented	972	64.7
Missing	812	5.6	Missing	77	5.1
**Age of mother at birth of child**					
20+	13284	91.1	20+	1386	92.3
Under 20	758	5.2	Under 20	85	5.7
Missing	543	3.7	Missing	31	2.1
**Smoking after 4 months preg.**					
Non smoker	9267	63.5	Non smoker	836	55.7
Smoker	4615	31.6	Smoker	617	41.1
Missing	703	4.8	Missing	49	3.3
**Ever breastfed**					
No	4257	29.2	No	629	41.9
Yes	9469	64.9	Yes	788	52.5
Missing	859	5.9	Missing	85	5.7
**Father’s role in child-rearing**					
Big, equal to mum	7832	53.7	Big, equal to mum	853	56.8
Dad sig, mum more	4062	27.9	Dad sig, mum more	349	23.2
Leaves mainly to mum	1448	9.9	Leaves mainly to mum	153	10.2
Missing	1243	8.5	Missing	147	9.8
**Frequency mum reads to child**					
Hardly ever	2178	14.9	Hardly ever	218	14.5
Occasionally	4791	32.8	Occasionally	509	33.9
Every week	6700	45.9	Every week	687	45.7
Missing	916	6.3	Missing	88	5.9
**Frequency dad reads to child**					
Hardly ever	3762	25.8	Hardly ever	352	23.4
Occasionally	4676	32.1	Occasionally	427	28.4
Every week	4783	32.8	Every week	564	37.5
Missing	1364	9.4	Missing	159	10.6
*Outcome variables*					
**Rutter scores**					
Normal	10689	73.3	Normal	1164	77.5
Moderate-severe	2258	15.5	Moderate-severe	208	13.8
Missing	1638	11.2	Missing	130	8.7
**Respiratory problems**					
No	12076	82.8	No	1271	84.6
Yes	2509	17.2	Yes	231	15.4
**Reading problems**					
No	11738	80.5	No	1214	80.8
Yes	2397	16.4	Yes	236	15.7
Missing	450	3.1	Missing	52	3.5

**Table 2:** **Descriptive statistics for relevant explanatory and outcome variables, BCS 1970**

	N	%		N	%
*Explanatory variables*					
**Area of residence (aged 5)**					
England	11157	90.5	GCV	377	30.2
Scotland	1166	9.5	Merseyside	420	33.7
			Gr. Manchester	450	36.1
**Social class of father**					
Class 1 & 2	1942	15.8	Class 1 & 2	130	10.4
Class 3	6907	56.0	Class 3	716	57.4
Class 4 & 5	2402	19.5	Class 4 & 5	325	26.1
Missing	1072	8.7	Missing	76	6.1
**Low birth weight**					
Not LBW	11215	91.0	Not LBW	1135	91.0
LBW	753	6.1	LBW	78	6.3
Missing	355	2.9	Missing	34	2.7
**Mother’s education**					
Some qualifications	5224	42.4	Some qualifications	468	37.5
No qualifications	6559	53.2	No qualifications	707	56.7
Missing	540	4.4	Missing	72	5.8
**Housing tenure**					
Owner occupied	6974	56.6	Owner occupied	644	51.6
Rented	5314	43.1	Rented	601	48.2
Missing	35	0.3	Missing	2	0.2
**Age of mother at birth of child**					
20+	10866	88.2	20+	1117	89.6
Under 20	1027	8.3	Under 20	106	8.5
Missing	430	3.5	Missing	24	1.9
**Smoking during pregnancy**					
Non smoker	7100	57.6	Non smoker	610	48.9
Smoker	4810	39.0	Smoker	618	49.6
Missing	413	3.4	Missing	19	1.5
**Ever breastfed**					
No	7575	61.5	No	885	71.0
Yes	4631	37.6	Yes	355	28.5
Missing	117	0.9	Missing	7	0.6
**Maternal mental health**					
Low-moderate malaise	9881	80.2	Low-moderate malaise	950	76.2
High malaise	2197	17.8	High malaise	270	21.7
Missing	245	2.0	Missing	27	2.2
**Dad helps mum put child to bed**					
Yes	10805	87.7	Yes	646	51.8
No	1192	9.7	No	548	43.9
Missing	326	2.6	Missing	53	4.3
**Who read to child in last week**					
Someone	10805	87.7	Someone	1062	85.2
No one	1192	9.7	No one	153	12.3
Missing	326	2.6	Missing	32	2.6
Missing			Missing		
*Outcome variables*					
**Rutter scores**					
Normal	9416	76.4	Normal	944	75.7
Moderate-severe	2260	18.3	Moderate-severe	248	19.9
Missing	647	5.3	Missing	55	4.4
**Respiratory problems**					
No	9823	79.7	No	1028	82.4
Yes	2475	20.1	Yes	215	17.2
Missing	25	0.2	Missing	4	0.3
**Vocabulary problems**					
No	11000	89.3	No	1112	89.2
Yes	1323	10.7	Yes	135	10.8

### Measures

#### Outcome measures

Three outcome measures, measured at age 7 in the NCDS and age 5 in the BCS70, were derived. These were behavioural problems, respiratory problems and reading/vocabulary problems. The measures were chosen because of their association with negative early years’ experiences and with subsequent risk of poor health and disadvantage in adulthood, as discussed above.

Behavioural problems were measured using Rutter scores at ages 5 (NCDS58) and 7 (BCS70). To derive these scores, cohort members’ mothers were asked a series of questions describing behaviour shown by many children and asked to what extent these applied to their own child (e.g. child ‘is miserable or tearful’, ‘is squirmy or fidgety’, ‘prefers to do things on his/her own rather than with other children’ (never/sometimes/frequently)). Responses to these questions were then combined into an index used to detect emotional/behavioural disturbances in children [30]. Using an approach described elsewhere [31], cohort members were classified as having normal (below the 80^th^ percentile), moderate (80^th^-95^th^ percentile) or severe (above the 95^th^ percentile) behavioural problems. This was then dichotomised into a simple normal *vs.* moderate-severe category. Respiratory problems were defined as the cohort member ever having an asthma attack or bronchitis with wheezing (NCDS58), following Strachan and Butland [32], or having a diagnosis of wheezing, asthma or bronchitis (BCS70). Cognitive ability was measured using the Southgate Group Reading Test (NCDS 1958) and the English Picture Vocabulary Test (BCS70). The Southgate Group Reading test was a measure of word recognition and comprehension. For 16 items, children were asked to look at a picture of an object and circle the word that picture represented; for a further 14, the teacher read out a word and children were again asked to circle the word that applied [33]. Reading problems in the NCDS58 were defined as scoring 0–15 (out of a possible 30) in the Southgate Group Reading Test at age 7 – one standard deviation below the mean. The English Picture Vocabulary Test was a measure of early English language development and understanding. Children were shown four pictures and a word was read out: they were asked to point to the picture which corresponded to the word being read out [34]. Vocabulary problems in the BCS70 were defined as scoring one standard deviation below the mean in the English Picture Vocabulary Test (EPVT) at age 5.

#### Explanatory variables

A range of explanatory variables were also used. Measures covered three themes (socio-economic status (SES), maternal health and parenting) and were selected based in prior research demonstrating their clear association with children’s health outcomes [6, 35]. SES measures included: father’s social class (used as an imperfect proxy for household income), child’s birth-weight, age of mother, mother’s education, housing tenure, age of mother at birth of the cohort member and (BCS70 only) family structure. Maternal health included measures of smoking in pregnancy, breastfeeding and (for the BCS70 only) maternal mental health. Parenting measures included measures of reading to the child and the role of the father in bringing up the cohort member. With the exception of social class, family structure, reading to child (NCDS only) and role of father (NCDS only), all explanatory variables were treated as dichotomous. Geographic variables showing country (Scotland/England) and region (Glasgow & the Clyde Valley/Greater Manchester/Merseyside) of residence were also added to the datasets. All explanatory variables were either measured at the same point in time as the outcome measure or shortly after the birth of the cohort member.

### Statistical analysis

In order to check the representativeness of the cohort studies, the social class distribution of these samples, at a national and city-region level, was compared with the 1971 and 1981 Censuses of Population. The social class distribution was found to be similar in both Censuses and cohort studies, increasing the likelihood that findings from these samples also apply to the broader population. Missing values were imputed using the multiple imputation option in SPSS 21. Multiple logistic regression was then used to measure the independent effect of nation and region of residence, on the three outcome measures. Scotland and Glasgow and the Clyde Valley were the reference categories for area of residence. Tables showing both the unadjusted and adjusted effect of area of residence on the three outcome variables were created [Tables 3 and 4]. The unadjusted figures show the effect of area of residence alone on the likelihood of having behavioural problems, respiratory problems or reading/vocabulary problems. Adjusted figures illustrate the effect of area of residence on the outcome measures after adjusting for SES, maternal health and parenting.

**Table 3:** **Odds ratio for moderate-high Rutter score, respiratory problems and reading problems by area of residence, missing values imputed, NCDS 1958**

***Area***	Odds ratio for moderate-high Rutter score		Odds ratio for respiratory problems		Odds ratio for reading problems	
Nation	***Unadjusted OR***	***Adjusted OR ǂ***	***Unadjusted OR***	***Adjusted OR ǂ***	***Unadjusted OR***	***Adjusted OR ǂ***
Scotland	1	1	1	1	1	1
England	1.32* (1.13 - 1.53)	1.36* (1.17 - 1.59)	1.50* (1.29 - 1.74)	1.58* (1.36 - 1.85)	2.01* (1.70 - 2.39)	2.57* (2.15 - 3.07)
	*p < 0.01*	*p < 0.01*	*p < 0.01*	*p < 0.01*	*P < 0.01*	*P < 0.01*
**City-region**						
GCV	1	1	1	1	1	1
Merseyside	1.88* (1.29 - 2.74)	1.75** (1.17 - 2.61)	1.60* (1.13 - 2.24)	1.70* (1.19 - 2.44)	2.00* (1.42 - 2.83)	2.34* (1.60 - 3.41)
Gr. Manchester	2.05* (1.43 - 2.94)	1.93* (1.32 - 2.84)	1.64* (1.16 - 2.30)	1.74* (1.21 - 2.51)	2.23* (1.59 - 3.13)	3.06* (2.10 - 4.46)

*p < 0.01 **p < 0.05. *ǂ* Adjusted for socio-economic circumstances, maternal health and parenting.

**Table 4:** **Odds ratio for moderate-high Rutter score, respiratory problems and vocabulary difficulties by area of residence, missing values imputed, BCS 1970**

***Area***	Odds ratio for moderate-high Rutter score		Odds ratio for respiratory problems		Odds ratio for vocabulary difficulties	
Nation	***Unadjusted OR***	***Adjusted OR ǂ***	***Unadjusted OR***	***Adjusted OR ǂ***	***Unadjusted OR***	***Adjusted OR ǂ***
Scotland	1	1	1	1	1	1
England	1.13 (0.96 - 1.33)	1.26** (1.06 -1.50)	1.24* (1.05 - 1.45)	1.37* (1.14 - 1.65)	0.89 (0.73 - 1.07)	0.95 (0.77 - 1.17)
	*P = NS*	*P < 0.01*	*p < 0.01*	*p < 0.01*	*p = NS*	*P < 0.01*
**City-region**						
GCV	1	1	1	1	1	1
Merseyside	1.12 (0.79 - 1.59)	0.80 (0.55 - 1.17)	1.07 (0.74 - 1.55)	1.10 (0.74 - 1.63)	0.68 (0.44 - 1.04)	0.66 (0.41 - 1.06)
Gr. Manchester	1.23 (0.87 - 1.73)	0.88 (0.63 - 1.24)	1.04 (0.72 - 1.49)	1.08 (0.73 - 1.60)	0.64 (0.42 - 0.99)	0.67 (0.41 - 1.07)

*p < 0.01 **p < 0.05. *ǂ* Adjusted for socio-economic circumstances, maternal health and parenting.

As a sensitivity analysis, the process was repeated for those cases for which information was complete for all variables. Results were similar, although the ‘complete cases’ approach produced slightly higher odds ratios and less precise confidence intervals.

## Results

In the NCDS58, cohort members resident in England at age 7 had an increased risk of behavioural problems (1.36, 1.17 to 1.59), respiratory problems (1.58, 1.36 to 1.85) and reading problems (2.57, 2.15 to 3.07) at age 7, even after adjustment for all other explanatory variables, compared with cohort members resident in Scotland. Cohort members resident in Merseyside or Greater Manchester at age 7 also had an increased risk of behavioural problems, respiratory problems and reading problems, compared with their GCV-resident peers (Table 3). This does not support the hypothesis that living in the Scottish areas is associated with a higher risk of poor childhood outcomes, once other factors are taken into account.

In the BCS70, cohort members resident in England at age 5 had an increased risk of behavioural problems (1.26, 1.06 to 1.50) and respiratory problems (1.37, 1.14 to 1.65) at age 5, after full adjustment, compared with cohort members resident in Scotland at the same age. Country of residence was not a significant predictor of vocabulary difficulties. Region of residence was not significantly associated with any of the three outcomes, after adjusting for SES, maternal health and parenting (Table 4). Again, this fails to confirm the hypothesis that living in Scottish areas was more detrimental to childhood outcomes, all other things being equal.

Taking both sets of results together, it appears that living in Scotland and GCV did not confer ‘excess’ behavioural problems, respiratory problems or reading/vocabulary problems in childhood. (Indeed, there is a suggestion that residence of Scotland may have provided some modest protective effects). It is difficult to see how EYCE which are better or no different might then translate into higher rates of poor health and mortality in adulthood. Other explanations might be required.

We can also look in more detail at the other determinants of childhood outcomes. At national level, socio-economic status, maternal health and parenting were all independently associated with the three outcomes. Parenting factors were relatively less important for respiratory problems. Predictors with the strongest association varied by outcome examined. For behaviour problems these included the father’s role in bringing up the child, age of mother, social class and mother’s mental health. For respiratory problems they included smoking in pregnancy, social class, mother’s mental health and low birth-weight. Finally, for reading/vocabulary difficulties these included reading to child, social class and low birth-weight.

Few variables had independent explanatory power at a regional level. This may reflect the similarities between the three regions. Maternal mental health was associated with all three outcomes (BCS70 cohort only), while reading/vocabulary problems were associated with lack of reading to the child and some indicators of socio-economic status (See Additional file 1).

## Discussion

Based on these two large, representative cohort study samples, the main finding of this study is that the evidence does not support the hypothesis that early years experiences (as measured here) were worse historically in Scotland and GCV, compared to England and Merseyside/Greater Manchester. After controlling for socio-economic status, maternal health and parenting measures, the childhood outcomes examined in the Scottish areas were either no different, or more favourable, compared to England and its two sub-regions.

The poor health profile in Scotland (and GCV) compared to other European countries is particularly driven by relatively high rates of female lung cancer, male suicide, chronic liver disease (including cirrhosis) and high rates of mortality among younger working-age adults (principally from external causes) [36]. This is relevant because of the links between EYCE and these health outcomes. For example, childhood behavioural problems appear to increase the risk of poor adult mental health, including Malaise and suicide, as well as the likelihood of smoking [15, 16, 37, 38]. However, given the results showed no clear excess in negative EYCE in the Scottish areas, they seems a less plausible pathway for increasing adult risk factors associated with the excess morbidity and mortality seen in the Scottish areas.

To the authors’ knowledge, this is the first study to test the hypothesis that living in Scotland and Glasgow in the 1960s and 1970s was associated with worse early years experiences and outcomes. It contributes to the existing literature on the ‘excess mortality’ seen in Scotland and GCV compared to England and comparable English cities and on the factors associated with early years’ outcomes. If they are taken at face value, the main findings suggest that key early years’ determinants of adult health were no worse in Scotland and GCV, compared to English areas, in the 1960s and 1970s. Unless the ‘dose response’ (for a population prevalence of behavioural problems, respiratory problems and reading/vocabulary problems) is higher in the Scottish areas, then EYCE seem a less plausible explanation for the poorer health and excess mortality seen relative to England, Greater Manchester and Merseyside.

One contemporary study provides some support for these findings. Dex (2008) used MCS data to explore a limited set of distinctive results for Scottish children born c. 2000/01. Among her findings, she concluded that after adjustment for background variables, risk of problem behaviour and (for one measure) cognitive development, was no different for Scottish children than those living in the rest of the UK [39]. Further confirmation for this is found in a recent survey of the three cities of Glasgow, Manchester and Liverpool, which asked respondents directly about how happy their childhood was and their relationship with their parents. It found little evidence on these measures that Glasgow childhoods were worse compared to the English cities [40]. The findings also reinforce the existing evidence on the combined influence of socio-economic factors, parental health and parenting factors on child outcomes, regardless of geography.

The study has important strengths. It is based on two large, representative samples which have been extensively used by social researchers. While several studies have explored the association between early years’ experiences and childhood health outcomes, few have included geography as an independent explanatory variable in this way and none have tested these outcomes at a city-region level. The study is one of the first to do so. It also confirms the important contribution that the combination of socio-economic status, maternal health and parenting can make to childhood outcomes. Even after controlling for parenting skills, material disadvantage still plays a role in determining early years’ outcomes [41]. On the other hand, poverty, by itself, does not necessarily lead to poor parenting [42, 43].

However, the study also has several limitations. Many of the measures rely on self-report by the parents (usually the mother), with results subject to both intentional (e.g. social desirability) and unintentional (e.g. recall) bias. This could be a particular issue as regards the Rutter scores. The gold standard of validation would be to compare responses to the same set of questions, on the same cohort of children, by parent, teacher and (if possible) child. More subtly, cultural norms could mean that parents from different backgrounds are answering questions in a different way. For example, despite their higher levels of mortality, the proportion of Scottish adults reporting they are in good-health is similar to England [44]. If something similar is also true for the Rutter scores, this could invalidate our assumptions. Lastly, since many of the outcomes and contextual measures were collected around the same point in time, we are unable to identify a causal link – the findings show association only.

The study also sheds little light on whether more extreme aspects of household dysfunction (such as abuse, neglect or parental substance misuse) were more likely to be experienced by children resident in the Scottish areas. It has been argued [28] that this is a plausible hypothesis, given higher levels of male imprisonment and opiate use in Scotland and GCV compared to England, Merseyside and Greater Manchester. The importance of this question was underlined by a recent qualitative study [45] conducted with recent Scottish drug injectors, which noted that many had been exposed to childhood trauma. However, this is difficult to test for directly. The NCDS58 and BCS70 cohort studies lack important measures of adverse childhood experiences, notably emotional neglect, sexual abuse and domestic violence. Parenting measures in these studies are much less comprehensive than those available in later cohorts. For example, the Millennium Cohort Survey uses the Pianta Child–parent Relationship Scale [46] to assess warmth and conflict between parent and child, which is not possible in earlier surveys. There are also some doubts about the comparability of measures of looked after children due to the different care systems that operate between countries. Perhaps most importantly, the high level of correlation between adverse childhood experiences (ACE) and disadvantage suggests that children exposed to these traumas are among the least likely to be included in population surveys and most likely to drop out through attrition.

In this context, one way forward might be to adapt the work by Kelly-Irving *et al*. [47], who used NCDS58 data to derive an adverse childhood experiences (ACE) variable and included it as a predictor of early mortality. While this approach has some limitations, it could be adopted to extend the present study. Such work could also exploit the longitudinal nature of the cohort studies, to investigate the association between early years’ experiences (including childhood health outcomes) and the determinants and outcomes of adult health in more detail. A second option might be conduct primary research based on the US ACE studies, in all four countries of the United Kingdom.

## Conclusions

This study does not support the hypothesis that early years’ experiences in general were worse in Scotland and GCV. Explanations for the excess poor health seen relative to England and comparable English city-regions may therefore lie elsewhere, though this does not exclude the possibility that more extreme aspects of family dysfunction may be at work in Scotland. This study also reinforces the need for a multifaceted approach for policy-makers interested in improving early years’ experiences, including as a means of improving adult health and reducing health inequalities. Regardless of geography, a combination of increasing families’ financial resources, improving parental health, especially maternal mental health, and supporting positive parenting (including ensuring fathers play an active role) remain vital to improving childhood outcomes.

## Electronic supplementary material

Additional file 1: **Twelve tables were also produced showing the independent explanatory power of all relevant variables (areas of residence, socio-economic characteristics, maternal health and parenting measures) in accounting for differences in adverse childhood experiences within multivariate models.** The tables present results from both cohort studies, using the Scotland/England and city-region samples separately. (DOCX 66 KB)
