# Supplementary material for: Comparing early years and childhood experiences and outcomes in Scotland, England and three city-regions: a plausible explanation for Scottish ‘excess’ mortality?
Source: BMC Pediatr. 2014 Oct 10;14:259. doi: 10.1186/1471-2431-14-259 (PMC4287510; doi:10.1186/1471-2431-14-259)
Supplement: Supplementary file 1 — Additional file 1: Twelve tables were also produced showing the independent explanatory power of all relevant variables (areas of residence, socio-economic characteristics, maternal health and parenting measures) in accounting for differences in adverse childhood experiences within multivariate models. The tables present results from both cohort studies, using the Scotland/England and city-region samples separately. (DOCX 66 KB) [file 12887_2014_1191_MOESM1_ESM.docx]

***Additional Tables***

*Table A1: Odds ratio for child having a moderate-high Rutter score at age 7, by area of residence, socio-economic characteristics, maternal health and parenting measures (imputed data): NCDS58, nations*

|  |  | **95% CI for Odds Ratio** | | |
| --- | --- | --- | --- | --- |
| Variable | β (s.e.) | Lower | Odds Ratio | Upper |
| Constant | -2.55 (0.14) |  |  |  |
| **Nation** |  |  |  |  |
| Scotland (ref) |  |  |  |  |
| England | 0.31 * (0.08) | 1.17 | 1.36 | 1.59 |
| **Father’s social class** |  |  |  |  |
| Social class I & II (ref) |  |  |  |  |
| Social class III | 0.20 ** (0.07) | 1.05 | 1.22 | 1.41 |
| Social class IV & V | 0.27 ** (0.09) | 1.08 | 1.31 | 1.58 |
| **Child birthweight** |  |  |  |  |
| Normal-high (ref) |  |  |  |  |
| Low | 0.06 (0.08) | 0.90 | 1.06 | 1.25 |
| **Mother education** |  |  |  |  |
| At school after MLA (ref) |  |  |  |  |
| Left school at MLA | 0.10 ǂ (0.06) | 0.98 | 1.11 | 1.25 |
| **Tenure** |  |  |  |  |
| Owner (ref) |  |  |  |  |
| Renter | 0.19 * (0.06) | 1.08 | 1.21 | 1.35 |
| **Age of mother** |  |  |  |  |
| Mother aged 20+ at birth of child (ref) |  |  |  |  |
| Mother aged <20 at birth of child | 0.45 * (0.09) | 1.32 | 1.57 | 1.87 |
| **Mother’s smoking in pregnancy** |  |  |  |  |
| Not smoking past 4 months (ref) |  |  |  |  |
| Smoking past 4 months | 0.22 * (0.05) | 1.13 | 1.24 | 1.37 |
| **Mother’s ever breastfed** |  |  |  |  |
| No (ref) |  |  |  |  |
| Yes | 0.18 * (0.05) | 1.08 | 1.20 | 1.33 |
| **Role of father in bringing up child** |  |  |  |  |
| Big role, equal to mum (ref) |  |  |  |  |
| Mum played more of a role, dad played a significant role | 0.20 * (0.05) | 1.10 | 1.22 | 1.36 |
| Mainly left to mum | 0.63 * (0.07) | 1.63 | 1.88 | 2.17 |
| **Mother reads to child** |  |  |  |  |
| Hardly ever (ref) |  |  |  |  |
| Occasionally | 0.08 (0.08) | 0.93 | 1.09 | 1.26 |
| Every week | -0.01 (0.07) | 0.86 | 0.99 | 1.15 |
| **Father reads to child** |  |  |  |  |
| Hardly ever (ref) |  |  |  |  |
| Occasionally | -0.09 (0.07) | 0.80 | 0.92 | 1.05 |
| Every week | -0.11 (0.08) | 0.77 | 0.90 | 1.06 |

p<0.01. *p<0.01, **p<0.05, ǂp<0.10.

*Table A2: Odds ratio for child having a moderate-high Rutter score at age 7, by area of residence, socio-economic characteristics, maternal health and parenting measures (imputed data): NCDS58, regions*

|  |  | **95% CI for Odds Ratio** | | |
| --- | --- | --- | --- | --- |
| Variable | β (s.e.) | Lower | Odds Ratio | Upper |
| Constant |  |  |  |  |
| **Nation** |  |  |  |  |
| Glasgow & the Clyde Valley |  |  |  |  |
| Merseyside | 0.56 ** (0.20) | 1.17 | 1.75 | 2.61 |
| Greater Manchester | 0.66 * (0.20) | 1.32 | 1.93 | 2.84 |
| **Father’s social class** |  |  |  |  |
| Social class I & II (ref) |  |  |  |  |
| Social class III | 0.15 (0.29) | 0.66 | 1.17 | 2.06 |
| Social class IV & V | 0.04 (0.34) | 0.53 | 1.04 | 2.07 |
| **Child birthweight** |  |  |  |  |
| Normal-high (ref) |  |  |  |  |
| Low | -0.13 (0.41) | 0.35 | 0.87 | 2.18 |
| **Mother education** |  |  |  |  |
| At school after MLA (ref) |  |  |  |  |
| Left school at MLA | 0.40 (0.26) | 0.90 | 1.49 | 2.47 |
| **Tenure** |  |  |  |  |
| Owner (ref) |  |  |  |  |
| Renter | 0.08 (0.19) | 0.75 | 1.08 | 1.56 |
| **Age of mother** |  |  |  |  |
| Mother aged 20+ at birth of child (ref) |  |  |  |  |
| Mother aged <20 at birth of child | 0.30 (0.31) | 0.73 | 1.35 | 2.50 |
| **Mother’s smoking in pregnancy** |  |  |  |  |
| Not smoking past 4 months (ref) |  |  |  |  |
| Smoking past 4 months | 0.06 (0.16) | 0.78 | 1.06 | 1.44 |
| **Mother’s ever breastfed** |  |  |  |  |
| No (ref) |  |  |  |  |
| Yes | 0.16 (0.16) | 0.85 | 1.18 | 1.63 |
| **Role of father in bringing up child** |  |  |  |  |
| Big role, equal to mum (ref) |  |  |  |  |
| Mum played more of a role, dad played a significant role | 0.08 (0.19) | 0.73 | 1.08 | 1.58 |
| Mainly left to mum | 0.61 (0.23) | 1.16 | 1.84 | 2.92 |
| **Mother reads to child** |  |  |  |  |
| Hardly ever (ref) |  |  |  |  |
| Occasionally | -0.19 (0.23) | 0.52 | 0.82 | 1.29 |
| Every week | -0.26 (0.24) | 0.48 | 0.77 | 1.23 |
| **Father reads to child** |  |  |  |  |
| Hardly ever (ref) |  |  |  |  |
| Occasionally | -0.29 (0.22) | 0.48 | 0.75 | 1.16 |
| Every week | -0.33 (0.28) | 0.40 | 0.72 | 1.28 |

p<0.01. *p<0.01, **p<0.05, ǂp<0.10.

*Table A3: Odds ratio for child having a moderate-high Rutter score at age 5, by area of residence, socio-economic characteristics, maternal health and parenting measures (imputed data): BCS70, nations*

|  |  | **95% CI for Odds Ratio** | | |
| --- | --- | --- | --- | --- |
| Variable | β (s.e.) | Lower | Odds Ratio | Upper |
| Constant | -3.72 (0.13) |  |  |  |
| **Nations** |  |  |  |  |
| Scotland (ref) |  |  |  |  |
| England | 0.23 ** (0.09) | 1.06 | 1.26 | 1.50 |
| **Father’s social class** |  |  |  |  |
| Social class I & II (ref) |  |  |  |  |
| Social class III | 0.26 * (0.09) | 1.10 | 1.30 | 1.54 |
| Social class IV & V | 0.31 * (0.10) | 1.13 | 1.37 | 1.66 |
| **Child birthweight** |  |  |  |  |
| Normal-high (ref) |  |  |  |  |
| Low | 0.18 ǂ (0.10) | 0.99 | 1.20 | 1.46 |
| **Mother education** |  |  |  |  |
| Some qualifications (ref) |  |  |  |  |
| No qualifications | 0.17 * (0.06) | 1.06 | 1.19 | 1.33 |
| **Tenure** |  |  |  |  |
| Owner (ref) |  |  |  |  |
| Renter | 0.19 * (0.05) | 1.08 | 1.21 | 1.34 |
| **Age of mother** |  |  |  |  |
| Mother aged 20+ at birth of child (ref) |  |  |  |  |
| Mother aged <20 at birth of child | 0.41 * (0.08) | 1.29 | 1.50 | 1.75 |
| **Family structure** |  |  |  |  |
| Natural father (ref) |  |  |  |  |
| Other father figure | 0.07 (0.13) | 0.83 | 1.07 | 1.39 |
| No father figure | 0.29 ** (0.11) | 1.08 | 1.34 | 1.66 |
| **Mothers mental health** |  |  |  |  |
| Malaise score 0-7 (ref) |  |  |  |  |
| Malaise score 8+ | 1.16 * (0.06) | 2.83 | 3.19 | 3.59 |
| **Mother’s smoking in pregnancy** |  |  |  |  |
| Never/gave up before or during preg. (ref) |  |  |  |  |
| Smoking during pregnancy | 0.23 * (0.05) | 1.14 | 1.26 | 1.39 |
| **Mother’s ever breastfed** |  |  |  |  |
| No (ref) |  |  |  |  |
| Yes | 0.08 (0.05) | 0.98 | 1.08 | 1.20 |
| **Role of father in bringing up child** |  |  |  |  |
| Father does not help put child to bed (ref) |  |  |  |  |
| Father helps put child to bed | -0.05 (0.07) | 0.81 | 0.95 | 1.11 |
| **Who reads to child regularly** |  |  |  |  |
| Somebody ref) |  |  |  |  |
| Nobody | 0.32 * (0.07) | 1.20 | 1.38 | 1.60 |

R^2^=.07 (Cox & Snell), .11 (Nagelkerke). Model X^2^ (1)=628.9, p<0.01, p; Model X^2^ (2)=895.9, p<0.01; Model X^2^ (3)=884.8, p<0.01; Model X^2^ (4)=855.0, p<0.01; Model X^2^ (5)=823.9, p<0.01; Model X^2^ (6)=854.5, p<0.01.

p<0.01. *p<0.01, **p<0.05, ǂp<0.10.

*Table A4: Odds ratio for child having a moderate-high Rutter score at age 5, by area of residence, socio-economic characteristics, maternal health and parenting measures (imputed data): BCS70, regions*

|  |  | **95% CI for Odds Ratio** | | |
| --- | --- | --- | --- | --- |
| Variable | β (s.e.) | Lower | Odds Ratio | Upper |
| Constant | -2.28 (0.33) |  |  |  |
| **Nations** |  |  |  |  |
| Glasgow and the Clyde Valley (ref) |  |  |  |  |
| Merseyside | -0.22 (0.19) | 0.55 | 0.80 | 1.17 |
| Greater Manchester | -0.13 (0.17) | 0.63 | 0.88 | 1.24 |
| **Father’s social class** |  |  |  |  |
| Social class I & II (ref) |  |  |  |  |
| Social class III | 0.38 (0.30) | 0.80 | 1.47 | 2.67 |
| Social class IV & V | 0.17 (0.33) | 0.71 | 1.36 | 2.58 |
| **Child birthweight** |  |  |  |  |
| Normal-high (ref) |  |  |  |  |
| Low | 0.44 (0.27) | 0.92 | 1.56 | 2.64 |
| **Mother education** |  |  |  |  |
| Some qualifications (ref) |  |  |  |  |
| No qualifications | 0.24 (0.17) | 0.91 | 1.27 | 1.78 |
| **Tenure** |  |  |  |  |
| Owner (ref) |  |  |  |  |
| Renter | 0.17 (0.18) | 0.83 | 1.18 | 1.69 |
| **Age of mother** |  |  |  |  |
| Mother aged 20+ at birth of child (ref) |  |  |  |  |
| Mother aged <20 at birth of child | 0.46 (0.23) | 1.00 | 1.58 | 2.50 |
| **Family structure** |  |  |  |  |
| Natural father (ref) |  |  |  |  |
| Other father figure | 0.14 (0.40) | 0.52 | 1.15 | 2.52 |
| No father figure | -0.10 (0.36) | 0.45 | 0.90 | 1.82 |
| **Mothers mental health** |  |  |  |  |
| Malaise score 0-7 (ref) |  |  |  |  |
| Malaise score 8+ | 0.97 * (0.16) | 1.92 | 2.64 | 3.63 |
| **Mother’s smoking in pregnancy** |  |  |  |  |
| Never/gave up before or during preg. (ref) |  |  |  |  |
| Smoking during pregnancy | 0.02 (0.16) | 0.75 | 1.02 | 1.39 |
| **Mother’s ever breastfed** |  |  |  |  |
| No (ref) |  |  |  |  |
| Yes | 0.14 (0.17) | 0.82 | 1.15 | 1.60 |
| **Role of father in bringing up child** |  |  |  |  |
| Father does not help put child to bed (ref) |  |  |  |  |
| Father helps put child to bed | 0.12 (0.17) | 0.81 | 1.13 | 1.57 |
| **Who reads to child regularly** |  |  |  |  |
| Somebody ref) |  |  |  |  |
| Nobody | 0.38 ǂ (0.21) | 0.96 | 1.46 | 2.23 |

R^2^=.06 (Cox & Snell), .09 (Nagelkerke). Model X^2^ (1)=56.3, p<0.01, p; Model X^2^ (2)=73.8, p<0.01; Model X^2^ (3)=76.5, p<0.01; Model X^2^ (4)=77.0, p<0.01; Model X^2^ (5)=73.9, p<0.01; Model X^2^ (6)=76.8, p<0.01. p<0.01. *p<0.01, **p<0.05, ǂp<0.10.

*Table A5: Odds ratio for child having a respiratory problem at age 7, by area of residence, socio-economic characteristics, maternal health and parenting measures (imputed data): NCDS58, nations*

|  |  | **95% CI for Odds Ratio** | | |
| --- | --- | --- | --- | --- |
| Variable | β (s.e.) | Lower | Odds Ratio | Upper |
| Constant | -2.22 (0.13) |  |  |  |
| **Nation** |  |  |  |  |
| Scotland (ref) |  |  |  |  |
| England | 0.46 * (0.08) | 1.36 | 1.58 | 1.85 |
| **Father’s social class** |  |  |  |  |
| Social class I & II (ref) |  |  |  |  |
| Social class III | -0.05 (0.07) | 0.82 | 0.95 | 1.10 |
| Social class IV & V | -0.06 (0.09) | 0.79 | 0.94 | 1.13 |
| **Child birthweight** |  |  |  |  |
| Normal-high (ref) |  |  |  |  |
| Low | -0.05 (0.09) | 0.79 | 0.95 | 1.14 |
| **Mother education** |  |  |  |  |
| At school after MLA (ref) |  |  |  |  |
| Left school at MLA | 0.08 (0.06) | 0.97 | 1.08 | 1.21 |
| **Tenure** |  |  |  |  |
| Owner (ref) |  |  |  |  |
| Renter | 0.08 (0.05) | 0.98 | 1.08 | 1.20 |
| **Age of mother** |  |  |  |  |
| Mother aged 20+ at birth of child (ref) |  |  |  |  |
| Mother aged <20 at birth of child | 0.32 * (0.09) | 1.15 | 1.38 | 1.66 |
| **Mother’s smoking in pregnancy** |  |  |  |  |
| Not smoking past 4 months (ref) |  |  |  |  |
| Smoking past 4 months | 0.21 * (0.05) | 1.12 | 1.23 | 1.35 |
| **Mother’s ever breastfed** |  |  |  |  |
| No (ref) |  |  |  |  |
| Yes | -0.05 (0.05) | 0.87 | 0.96 | 1.05 |
| **Role of father in bringing up child** |  |  |  |  |
| Big role, equal to mum (ref) |  |  |  |  |
| Mum played more of a role, dad played a significant role | 0.09 ǂ (0.05) | 0.99 | 1.09 | 1.21 |
| Mainly left to mum | 0.08 (0.08) | 0.93 | 1.08 | 1.26 |
| **Mother reads to child** |  |  |  |  |
| Hardly ever (ref) |  |  |  |  |
| Occasionally | 0.18 ** (0.07) | 1.04 | 1.19 | 1.38 |
| Every week | 0.17 ** (0.08) | 1.02 | 1.18 | 1.37 |
| **Father reads to child** |  |  |  |  |
| Hardly ever (ref) |  |  |  |  |
| Occasionally | -0.15 ** (0.06) | 0.76 | 0.86 | 0.97 |
| Every week | -0.03 (0.07) | 0.85 | 0.97 | 1.11 |

p<0.01. *p<0.01, **p<0.05, ǂp<0.10.

*Table A6: Odds ratio for child having a respiratory problem at age 7, by area of residence, socio-economic characteristics, maternal health and parenting measures (imputed data): NCDS58, regions*

|  |  | **95% CI for Odds Ratio** | | |
| --- | --- | --- | --- | --- |
| Variable | β (s.e.) | Lower | Odds Ratio | Upper |
| Constant | -2.57 (0.38) |  |  |  |
| **Nation** |  |  |  |  |
| Glasgow & the Clyde Valley |  |  |  |  |
| Merseyside | 0.53 * (0.18) | 1.19 | 1.70 | 2.44 |
| Greater Manchester | 0.55 * (0.19) | 1.21 | 1.74 | 2.51 |
| **Father’s social class** |  |  |  |  |
| Social class I & II (ref) |  |  |  |  |
| Social class III | 0.19 (0.25) | 0.74 | 1.21 | 1.98 |
| Social class IV & V | 0.08 (0.29) | 0.62 | 1.09 | 1.90 |
| **Child birthweight** |  |  |  |  |
| Normal-high (ref) |  |  |  |  |
| Low | -0.29 (0.31) | 0.40 | 0.75 | 1.42 |
| **Mother education** |  |  |  |  |
| At school after MLA (ref) |  |  |  |  |
| Left school at MLA | 0.17 (0.23) | 0.76 | 1.19 | 1.85 |
| **Tenure** |  |  |  |  |
| Owner (ref) |  |  |  |  |
| Renter | -0.17 (0.18) | 0.59 | 0.84 | 1.21 |
| **Age of mother** |  |  |  |  |
| Mother aged 20+ at birth of child (ref) |  |  |  |  |
| Mother aged <20 at birth of child | -0.52 (0.37) | 0.29 | 0.59 | 1.23 |
| **Mother’s smoking in pregnancy** |  |  |  |  |
| Not smoking past 4 months (ref) |  |  |  |  |
| Smoking past 4 months | 0.26 (0.15) | 0.96 | 1.30 | 1.75 |
| **Mother’s ever breastfed** |  |  |  |  |
| No (ref) |  |  |  |  |
| Yes | -0.25 (0.15) | 0.58 | 0.78 | 1.05 |
| **Role of father in bringing up child** |  |  |  |  |
| Big role, equal to mum (ref) |  |  |  |  |
| Mum played more of a role, dad played a significant role | 0.16 (0.19) | 0.82 | 1.18 | 1.70 |
| Mainly left to mum | -0.08 (0.27) | 0.54 | 0.92 | 1.57 |
| **Mother reads to child** |  |  |  |  |
| Hardly ever (ref) |  |  |  |  |
| Occasionally | 0.31 (0.29) | 0.76 | 1.37 | 2.45 |
| Every week | 0.29 (0.29) | 0.75 | 1.33 | 2.38 |
| **Father reads to child** |  |  |  |  |
| Hardly ever (ref) |  |  |  |  |
| Occasionally | 0.17 (0.29) | 0.64 | 1.19 | 2.21 |
| Every week | 0.26 (0.27) | 0.74 | 1.30 | 2.25 |

p<0.01. *p<0.01, **p<0.05, ǂp<0.10.

*Table A7: Odds ratio for child having a respiratory problem at age 5, by area of residence, socio-economic characteristics, maternal health and parenting measures (imputed data): BCS70, nations*

|  |  | **95% CI for Odds Ratio** | | |
| --- | --- | --- | --- | --- |
| Variable | β (s.e.) | Lower | Odds Ratio | Upper |
| Constant | -2.15 (0.14) |  |  |  |
| **Nations** |  |  |  |  |
| Scotland (ref) |  |  |  |  |
| England | 0.31 * (0.10) | 1.14 | 1.37 | 1.65 |
| **Father’s social class** |  |  |  |  |
| Social class I & II (ref) |  |  |  |  |
| Social class III | 0.03 (0.07) | 0.89 | 1.03 | 1.19 |
| Social class IV & V | 0.02 (0.09) | 0.85 | 1.02 | 1.22 |
| **Child birthweight** |  |  |  |  |
| Normal-high (ref) |  |  |  |  |
| Low | 0.23 ** (0.10) | 1.03 | 1.26 | 1.53 |
| **Mother education** |  |  |  |  |
| Some qualifications (ref) |  |  |  |  |
| No qualifications | -0.05 (0.06) | 0.85 | 0.95 | 1.06 |
| **Tenure** |  |  |  |  |
| Owner (ref) |  |  |  |  |
| Renter | 0.09 (0.06) | 0.98 | 1.10 | 1.23 |
| **Age of mother** |  |  |  |  |
| Mother aged 20+ at birth of child (ref) |  |  |  |  |
| Mother aged <20 at birth of child | -0.04 (0.10) | 0.79 | 0.96 | 1.17 |
| **Family structure** |  |  |  |  |
| Natural father (ref) |  |  |  |  |
| Other father figure | 0.06 (0.16) | 0.77 | 1.06 | 1.46 |
| No father figure | 0.15 (0.13) | 0.90 | 1.16 | 1.50 |
| **Mothers mental health** |  |  |  |  |
| Malaise score 0-7 (ref) |  |  |  |  |
| Malaise score 8+ | 0.31 * (0.07) | 1.20 | 1.37 | 1.55 |
| **Mother’s smoking in pregnancy** |  |  |  |  |
| Never/gave up before or during preg. (ref) |  |  |  |  |
| Smoking during pregnancy | 0.27 * (0.05) | 1.18 | 1.31 | 1.46 |
| **Mother’s ever breastfed** |  |  |  |  |
| No (ref) |  |  |  |  |
| Yes | -0.10 ǂ (0.05) | 0.82 | 0.91 | 1.01 |
| **Role of father in bringing up child** |  |  |  |  |
| Father does not help put child to bed (ref) |  |  |  |  |
| Father helps put child to bed | 0.05 (0.05) | 0.95 | 1.05 | 1.16 |
| **Who reads to child regularly** |  |  |  |  |
| Somebody ref) |  |  |  |  |
| Nobody | -0.08 (0.09) | 0.77 | 0.92 | 1.10 |

p<0.01. *p<0.01, **p<0.05, ǂp<0.10.

*Table A8: Odds ratio for child having a respiratory problem at age 5, by area of residence, socio-economic characteristics, maternal health and parenting measures (imputed data): BCS70, regions*

|  |  | **95% CI for Odds Ratio** | | |
| --- | --- | --- | --- | --- |
| Variable | β (s.e.) | Lower | Odds Ratio | Upper |
| Constant | -2.08 (0.34) |  |  |  |
| **Nations** |  |  |  |  |
| Glasgow and the Clyde Valley (ref) |  |  |  |  |
| Merseyside | 0.10 (0.20) | 0.74 | 1.10 | 1.63 |
| Greater Manchester | 0.08 (0.20) | 0.73 | 1.08 | 1.60 |
| **Father’s social class** |  |  |  |  |
| Social class I & II (ref) |  |  |  |  |
| Social class III | -0.08 (0.26) | 0.55 | 0.93 | 1.55 |
| Social class IV & V | 0.04 (0.29) | 0.59 | 1.05 | 1.86 |
| **Child birthweight** |  |  |  |  |
| Normal-high (ref) |  |  |  |  |
| Low | -0.52 (0.35) | 0.30 | 0.59 | 1.19 |
| **Mother education** |  |  |  |  |
| Some qualifications (ref) |  |  |  |  |
| No qualifications | -0.01 (0.18) | 0.69 | 0.99 | 1.40 |
| **Tenure** |  |  |  |  |
| Owner (ref) |  |  |  |  |
| Renter | -0.05 (0.18) | 0.66 | 0.95 | 1.35 |
| **Age of mother** |  |  |  |  |
| Mother aged 20+ at birth of child (ref) |  |  |  |  |
| Mother aged <20 at birth of child | 0.41 (0.25) | 0.92 | 1.50 | 2.45 |
| **Family structure** |  |  |  |  |
| Natural father (ref) |  |  |  |  |
| Other father figure | 0.25 (0.40) | 0.59 | 1.29 | 2.80 |
| No father figure | 0.02 (0.35) | 0.52 | 1.02 | 2.02 |
| **Mothers mental health** |  |  |  |  |
| Malaise score 0-7 (ref) |  |  |  |  |
| Malaise score 8+ | 0.38 ** (0.18) | 1.03 | 1.46 | 2.09 |
| **Mother’s smoking in pregnancy** |  |  |  |  |
| Never/gave up before or during preg. (ref) |  |  |  |  |
| Smoking during pregnancy | 0.27 (0.16) | 0.95 | 1.31 | 1.80 |
| **Mother’s ever breastfed** |  |  |  |  |
| No (ref) |  |  |  |  |
| Yes | -0.33 ǂ (0.19) | 0.50 | 0.72 | 1.04 |
| **Role of father in bringing up child** |  |  |  |  |
| Father does not help put child to bed (ref) |  |  |  |  |
| Father helps put child to bed | -0.08 (0.16) | 0.68 | 0.93 | 1.27 |
| **Who reads to child regularly** |  |  |  |  |
| Somebody ref) |  |  |  |  |
| Nobody | 0.02 (0.23) | 0.65 | 1.02 | 1.59 |

p<0.01. *p<0.01, **p<0.05, ǂp<0.10.

*Table A9: Odds ratio for child having reading problems at age 7, by area of residence, socio-economic characteristics, maternal health and parenting measures (imputed data): NCDS58, nations*

|  |  | **95% CI for Odds Ratio** | | |
| --- | --- | --- | --- | --- |
| Variable | β (s.e.) | Lower | Odds Ratio | Upper |
| Constant | -3.48 (0.15) |  |  |  |
| **Nation** |  |  |  |  |
| Scotland (ref) |  |  |  |  |
| England | 0.94 * (0.09) | 2.15 | 2.57 | 3.07 |
| **Father’s social class** |  |  |  |  |
| Social class I & II (ref) |  |  |  |  |
| Social class III | 0.56 * (0.10) | 1.44 | 1.74 | 2.11 |
| Social class IV & V | 0.90 * (0.10) | 2.01 | 2.46 | 3.01 |
| **Child birthweight** |  |  |  |  |
| Normal-high (ref) |  |  |  |  |
| Low | 0.38 * (0.09) | 1.23 | 1.47 | 1.74 |
| **Mother education** |  |  |  |  |
| At school after MLA (ref) |  |  |  |  |
| Left school at MLA | 0.60 * (0.08) | 1.56 | 1.82 | 2.14 |
| **Tenure** |  |  |  |  |
| Owner (ref) |  |  |  |  |
| Renter | 0.56 * (0.07) | 1.53 | 1.75 | 2.01 |
| **Age of mother** |  |  |  |  |
| Mother aged 20+ at birth of child (ref) |  |  |  |  |
| Mother aged <20 at birth of child | 0.25 ** (0.09) | 1.07 | 1.28 | 1.55 |
| **Mother’s smoking in pregnancy** |  |  |  |  |
| Not smoking past 4 months (ref) |  |  |  |  |
| Smoking past 4 months | 0.16 * (0.05) | 1.06 | 1.17 | 1.29 |
| **Mother’s ever breastfed** |  |  |  |  |
| No (ref) |  |  |  |  |
| Yes | -0.22 * (0.05) | 0.72 | 0.80 | 0.89 |
| **Role of father in bringing up child** |  |  |  |  |
| Big role, equal to mum (ref) |  |  |  |  |
| Mum played more of a role, dad played a significant role | -0.09 (0.06) | 0.81 | 0.91 | 1.02 |
| Mainly left to mum | 0.26 * (0.08) | 1.11 | 1.30 | 1.53 |
| **Mother reads to child** |  |  |  |  |
| Hardly ever (ref) |  |  |  |  |
| Occasionally | -0.14 ** (0.07) | 0.76 | 0.87 | 0.99 |
| Every week | -0.13 ǂ (0.07) | 0.76 | 0.88 | 1.01 |
| **Father reads to child** |  |  |  |  |
| Hardly ever (ref) |  |  |  |  |
| Occasionally | -0.32 * (0.06) | 0.64 | 0.72 | 0.82 |
| Every week | -0.47 * (0.07) | 0.55 | 0.63 | 0.72 |

*p<0.01, **p<0.05, ǂp<0.10.

*Table A10: Odds ratio for child having reading problems at age 7, by area of residence, socio-economic characteristics, maternal health and parenting measures (imputed data): NCDS58, regions*

|  |  | **95% CI for Odds Ratio** | | |
| --- | --- | --- | --- | --- |
| Variable | β (s.e.) | Lower | Odds Ratio | Upper |
| Constant | -3.21 (0.47) |  |  |  |
| **Nation** |  |  |  |  |
| Glasgow & the Clyde Valley |  |  |  |  |
| Merseyside | 0.85 * (0.19) | 1.60 | 2.34 | 3.41 |
| Greater Manchester | 1.12 * (0.19) | 2.10 | 3.06 | 4.46 |
| **Father’s social class** |  |  |  |  |
| Social class I & II (ref) |  |  |  |  |
| Social class III | 0.67 (0.38) | 0.92 | 1.95 | 4.12 |
| Social class IV & V | 1.06 ** (0.38) | 1.37 | 2.90 | 6.14 |
| **Child birthweight** |  |  |  |  |
| Normal-high (ref) |  |  |  |  |
| Low | 0.21 (0.24) | 0.76 | 1.24 | 2.00 |
| **Mother education** |  |  |  |  |
| At school after MLA (ref) |  |  |  |  |
| Left school at MLA | 0.30 (0.28) | 0.77 | 1.34 | 2.35 |
| **Tenure** |  |  |  |  |
| Owner (ref) |  |  |  |  |
| Renter | 0.50 ** (0.20) | 1.11 | 1.64 | 2.44 |
| **Age of mother** |  |  |  |  |
| Mother aged 20+ at birth of child (ref) |  |  |  |  |
| Mother aged <20 at birth of child | 0.88 * (0.27) | 1.41 | 2.41 | 4.12 |
| **Mother’s smoking in pregnancy** |  |  |  |  |
| Not smoking past 4 months (ref) |  |  |  |  |
| Smoking past 4 months | 0.40 ** (0.16) | 1.08 | 1.49 | 2.04 |
| **Mother’s ever breastfed** |  |  |  |  |
| No (ref) |  |  |  |  |
| Yes | -0.70 * (0.17) | 0.35 | 0.50 | 0.70 |
| **Role of father in bringing up child** |  |  |  |  |
| Big role, equal to mum (ref) |  |  |  |  |
| Mum played more of a role, dad played a significant role | -0.11 (0.21) | 0.59 | 0.89 | 1.35 |
| Mainly left to mum | 0.41 (0.26) | 0.90 | 1.51 | 2.52 |
| **Mother reads to child** |  |  |  |  |
| Hardly ever (ref) |  |  |  |  |
| Occasionally | -0.16 (0.25) | 0.52 | 0.85 | 1.41 |
| Every week |  | 0.51 | 0.86 | 1.44 |
| **Father reads to child** |  |  |  |  |
| Hardly ever (ref) |  |  |  |  |
| Occasionally | -0.15 (0.26) | 0.67 | 0.99 | 1.45 |
| Every week | -0.01 ** (0.20) | 0.37 | 0.58 | 0.93 |

p<0.01. *p<0.01, **p<0.05, ǂp<0.10.

*Table A11: Odds ratio for child having vocabulary problems at age 5, by area of residence, socio-economic characteristics, maternal health and parenting measures (imputed data): BCS70, nations*

|  |  | **95% CI for Odds Ratio** | | |
| --- | --- | --- | --- | --- |
| Variable | β (s.e.) | Lower | Odds Ratio | Upper |
| Constant | -2.80 (0.16) |  |  |  |
| **Nations** |  |  |  |  |
| Scotland (ref) |  |  |  |  |
| England | -0.05 (0.11) | 0.77 | 0.95 | 1.17 |
| **Father’s social class** |  |  |  |  |
| Social class I & II (ref) |  |  |  |  |
| Social class III | 0.03 (0.10) | 0.85 | 1.03 | 1.25 |
| Social class IV & V | 0.42 * (0.11) | 1.22 | 1.52 | 1.90 |
| **Child birthweight** |  |  |  |  |
| Normal-high (ref) |  |  |  |  |
| Low | 0.57 * (0.12) | 1.40 | 1.76 | 2.21 |
| **Mother education** |  |  |  |  |
| Some qualifications (ref) |  |  |  |  |
| No qualifications | 0.40 * (0.07) | 1.29 | 1.49 | 1.72 |
| **Tenure** |  |  |  |  |
| Owner (ref) |  |  |  |  |
| Renter | 0.10 (0.07) | 0.97 | 1.11 | 1.27 |
| **Age of mother** |  |  |  |  |
| Mother aged 20+ at birth of child (ref) |  |  |  |  |
| Mother aged <20 at birth of child | 0.33 * (0.10) | 1.14 | 1.40 | 1.71 |
| **Family structure** |  |  |  |  |
| Natural father (ref) |  |  |  |  |
| Other father figure | -0.48 ** (0.22) | 0.41 | 0.62 | 0.94 |
| No father figure | 0.03 (0.15) | 0.77 | 1.03 | 1.38 |
| **Mothers mental health** |  |  |  |  |
| Malaise score 0-7 (ref) |  |  |  |  |
| Malaise score 8+ | 0.27 * (0.08) | 1.12 | 1.31 | 1.52 |
| **Mother’s smoking in pregnancy** |  |  |  |  |
| Never/gave up before or during preg. (ref) |  |  |  |  |
| Smoking during pregnancy | -0.23 * (0.07) | 0.70 | 0.79 | 0.91 |
| **Mother’s ever breastfed** |  |  |  |  |
| No (ref) |  |  |  |  |
| Yes | 0.06 (0.07) | 0.93 | 1.06 | 1.21 |
| **Role of father in bringing up child** |  |  |  |  |
| Father does not help put child to bed (ref) |  |  |  |  |
| Father helps put child to bed | -0.26 * (0.07) | 0.68 | 0.77 | 0.88 |
| **Who reads to child regularly** |  |  |  |  |
| Somebody ref) |  |  |  |  |
| Nobody | 0.65 * (0.09) | 1.60 | 1.91 | 2.28 |

p<0.01. *p<0.01, **p<0.05, ǂp<0.10.

*Table A12: Odds ratio for child having vocabulary problems at age 5, by area of residence, socio-economic characteristics, maternal health and parenting measures (imputed data): BCS70, regions*

|  |  | **95% CI for Odds Ratio** | | |
| --- | --- | --- | --- | --- |
| Variable | β (s.e.) | Lower | Odds Ratio | Upper |
| Constant | -3.24 (0.47) |  |  |  |
| **Nations** |  |  |  |  |
| Glasgow and the Clyde Valley (ref) |  |  |  |  |
| Merseyside | -0.42 ǂ (0.24) | 0.41 | 0.66 | 1.06 |
| Greater Manchester | -0.41 ǂ (0.24) | 0.41 | 0.67 | 1.07 |
| **Father’s social class** |  |  |  |  |
| Social class I & II (ref) |  |  |  |  |
| Social class III | 0.15 (0.39) | 0.54 | 1.17 | 2.53 |
| Social class IV & V | 0.33 (0.43) | 0.60 | 1.38 | 3.19 |
| **Child birthweight** |  |  |  |  |
| Normal-high (ref) |  |  |  |  |
| Low | 0.92 * (0.31) | 1.36 | 2.52 | 4.65 |
| **Mother education** |  |  |  |  |
| Some qualifications (ref) |  |  |  |  |
| No qualifications | 0.52 ** (0.24) | 1.05 | 1.69 | 2.69 |
| **Tenure** |  |  |  |  |
| Owner (ref) |  |  |  |  |
| Renter | 0.04 (0.23) | 1.05 | 1.69 | 2.69 |
| **Age of mother** |  |  |  |  |
| Mother aged 20+ at birth of child (ref) |  |  |  |  |
| Mother aged <20 at birth of child | 0.19 (0.31) | 0.65 | 1.21 | 2.24 |
| **Family structure** |  |  |  |  |
| Natural father (ref) |  |  |  |  |
| Other father figure | -0.52 (0.64) | 0.17 | 0.59 | 2.07 |
| No father figure | 0.21 (0.40) | 0.56 | 1.23 | 2.69 |
| **Mothers mental health** |  |  |  |  |
| Malaise score 0-7 (ref) |  |  |  |  |
| Malaise score 8+ | 0.57 ** (0.21) | 1.17 | 1.76 | 2.66 |
| **Mother’s smoking in pregnancy** |  |  |  |  |
| Never/gave up before or during preg. (ref) |  |  |  |  |
| Smoking during pregnancy | -0.08 (0.20) | 0.63 | 0.93 | 1.38 |
| **Mother’s ever breastfed** |  |  |  |  |
| No (ref) |  |  |  |  |
| Yes | -0.08 (0.24) | 0.58 | 0.92 | 1.46 |
| **Role of father in bringing up child** |  |  |  |  |
| Father does not help put child to bed (ref) |  |  |  |  |
| Father helps put child to bed | -0.08 (0.20) | 0.63 | 0.92 | 1.36 |
| **Who reads to child regularly** |  |  |  |  |
| Somebody ref) |  |  |  |  |
| Nobody | 0.61 ** (0.24) | 1.15 | 1.83 | 2.93 |

*p<0.01, **p<0.05, ǂp<0.10.
